# Supplementary material for: Redox-Responsive Lipidic Prodrug Nano-Delivery System Improves Antitumor Effect of Curcumin Derivative C210
Source: Pharmaceutics. 2023 May 19;15(5):1546. doi: 10.3390/pharmaceutics15051546 (PMC10220537; doi:10.3390/pharmaceutics15051546)
Supplement: Supplementary file 1 [file pharmaceutics-15-01546-s001.zip › pharmaceutics-2368317-supplementary.pdf]

Supplementary Material

Redox-Responsive Lipidic Prodrug Nano-Delivery System Improves Antitumor Effect of Curcumin Derivative C210

Xin Guo [1,2], Min Wu [1,2], Yanping Deng [1,2,*], Yan Liu [1,2], Yanpeng Liu [1,2] and Jianhua Xu [1,2,*]

[1] The School of Pharmacy, Fujian Medical University, Fuzhou 350122, China; 17705005241@163.com (X.G.); 18450056606@163.com (M.W.); 13959147061@163.com (Y.L.); 15617186956@163.com (Y.L.)
[2] Fujian Provincial Key Laboratory of Natural Medicine Pharmacology, Fujian Medical University, Fuzhou 350122, China
* Correspondence: yanpingdeng@fjmu.edu.cn (Y.D.); xjh@fjmu.edu.cn (J.X.)

Characterization of C210-R-OA prodrugs

2-methoxy-4-((E)-3-oxo-5-(3,4,5-trimethoxyphenyl)-2-((E)-3-(3,4,5-trimethoxyphenyl) acryloyl)pent-4-en-1-yl)phenyl 2-((2-(((E)-octadec-9-en-1-yl)oxy)-2-oxoethyl)thio)acetate. (C210-S-OA)

TOF MS (ES-): calcd. for $[C_{55}H_{74}O_{13}S\text{-}H]^-$ 973.4772, found 973.4774.

^{1}H NMR (500 MHz, $CDCl_3$) δ 7.69 (d, J = 15.3 Hz, 2H), 6.93 – 6.86 (m, 2H), 6.83 (d, J = 15.3 Hz, 2H), 6.67 (s, 3H), 5.35 (dqd, J = 7.9, 4.6, 4.0, 2.1 Hz, 2H), 4.13 (t, J = 6.9 Hz, 2H), 3.98 (s, 2H), 3.93 – 3.82 (m, 19H), 3.77 (s, 3H), 3.62 (d, J = 5.6 Hz, 2H), 3.48 (s, 2H), 2.06 – 1.97 (m, 4 H), 1.64 (t, J = 7.3 Hz, 2H), 1.58 (s, 2H), 1.35 – 1.22 (m, 24H), 0.88 (t, J = 6.8 Hz, 3H).

^{13}C NMR (126 MHz, $CDCl_3$) δ 183.56, 169.82, 168.05, 153.44, 151.27, 140.40, 140.20, 138.17, 130.72, 130.01, 129.76, 122.79, 119.92, 111.82, 108.68, 105.92, 105.40, 65.75, 61.00, 56.24, 56.19, 55.90, 33.39, 33.23, 31.91, 31.78, 29.77, 29.72, 29.53, 29.40, 29.32, 29.20, 28.53, 27.22, 27.18, 25.81, 22.69, 14.13.

2-methoxy-4-((E)-3-oxo-5-(3,4,5-trimethoxyphenyl)-2-((E)-3-(3,4,5-trimethoxyphenyl)acryloyl)pent-4-en-1-yl)phenyl 2-((2-(((E)-octadec-9-en-1-yl)oxy)-2-oxoethyl)disulfaneyl)acetate. (C210-SS-OA)

TOF MS (ES-): calcd. for $[C_{55}H_{74}O_{13}S_2\text{-}H]^-$ 1005.4493, found 1005.4497.

^{1}H NMR (500 MHz, $CDCl_3$) δ 7.69 (d, J = 15.3 Hz, 2H), 6.93 – 6.80 (m, 4H), 6.67 (s, 3H), 5.39 – 5.30 (m, 2H), 4.13 (dd, J = 7.3, 6.2 Hz, 2H), 3.98 (s, 2H), 3.91 – 3.76 (m, 23H), 3.62 (s, 2H), 2.06 – 1.92 (m, 4H), 1.68 – 1.60 (m, 3H), 1.36 – 1.23 (m, 24H), 0.88 (t, J = 6.9 Hz, 3H).

^{13}C NMR (126 MHz, CDCl3) δ 183.56, 169.38, 167.68, 153.44, 151.33, 142.26, 140.45, 140.19, 138.18, 130.72, 130.00, 129.77, 122.82, 119.93, 119.89, 111.85, 108.69, 105.40, 65.90, 61.02, 61.00, 56.19, 55.90, 55.86, 41.44, 41.03, 31.91, 29.77, 29.73, 29.53, 29.41, 29.33, 29.21, 29.18, 28.51, 27.22, 27.19, 25.81, 22.69, 14.13.

2-methoxy-4-((E)-3-oxo-5-(3,4,5-trimethoxyphenyl)-2-((E)-3-(3,4,5-trimethoxyphenyl)acryloyl)pent-4-en-1-yl)phenyl ((E)-octadec-9-en-1-yl) glutarate. (C210-C-OA)

TOF MS (ES-): calcd. for $[C_{56}H_{76}O_{13}\text{-}H]^-$ 955.5208, found 955.5206.

^{1}H NMR (500 MHz, $CDCl_3$) δ 7.69 (d, J = 15.3 Hz, 2H), 6.93 – 6.80 (m, 4H), 6.67 (s, 3H), 5.40 – 5.27 (m, 2H), 4.08 (t, J = 6.8 Hz, 2H), 3.97 (s, 2H), 3.86 (d, J = 6.0 Hz, 18H), 3.76 (s, 3H), 2.64 (t, J = 7.2 Hz, 2H), 2.46 (t, J = 7.4 Hz, 2H), 2.11 – 2.03 (m, 2H), 2.03 – 1.93 (m, 4H), 1.62 (t, J = 7.2 Hz, 2H), 1.37 – 1.22 (m, 25H), 0.88 (t, J = 6.9 Hz, 3H).

^{13}C NMR (126 MHz, $CDCl_3$) δ 183.56, 172.99, 171.20, 153.46, 153.43, 151.38, 142.19, 140.16, 140.04, 138.32, 130.74, 129.98, 129.77, 122.92, 119.98, 119.87, 111.74, 108.78, 105.39, 64.70, 64.68, 60.98, 56.22, 56.17, 55.83, 55.80, 33.12, 33.06, 31.90, 31.76, 29.76, 29.72, 29.70, 29.65, 29.52, 29.41, 29.32, 29.23, 29.21, 28.63, 27.21, 27.18, 25.90, 22.68, 20.29, 14.12.

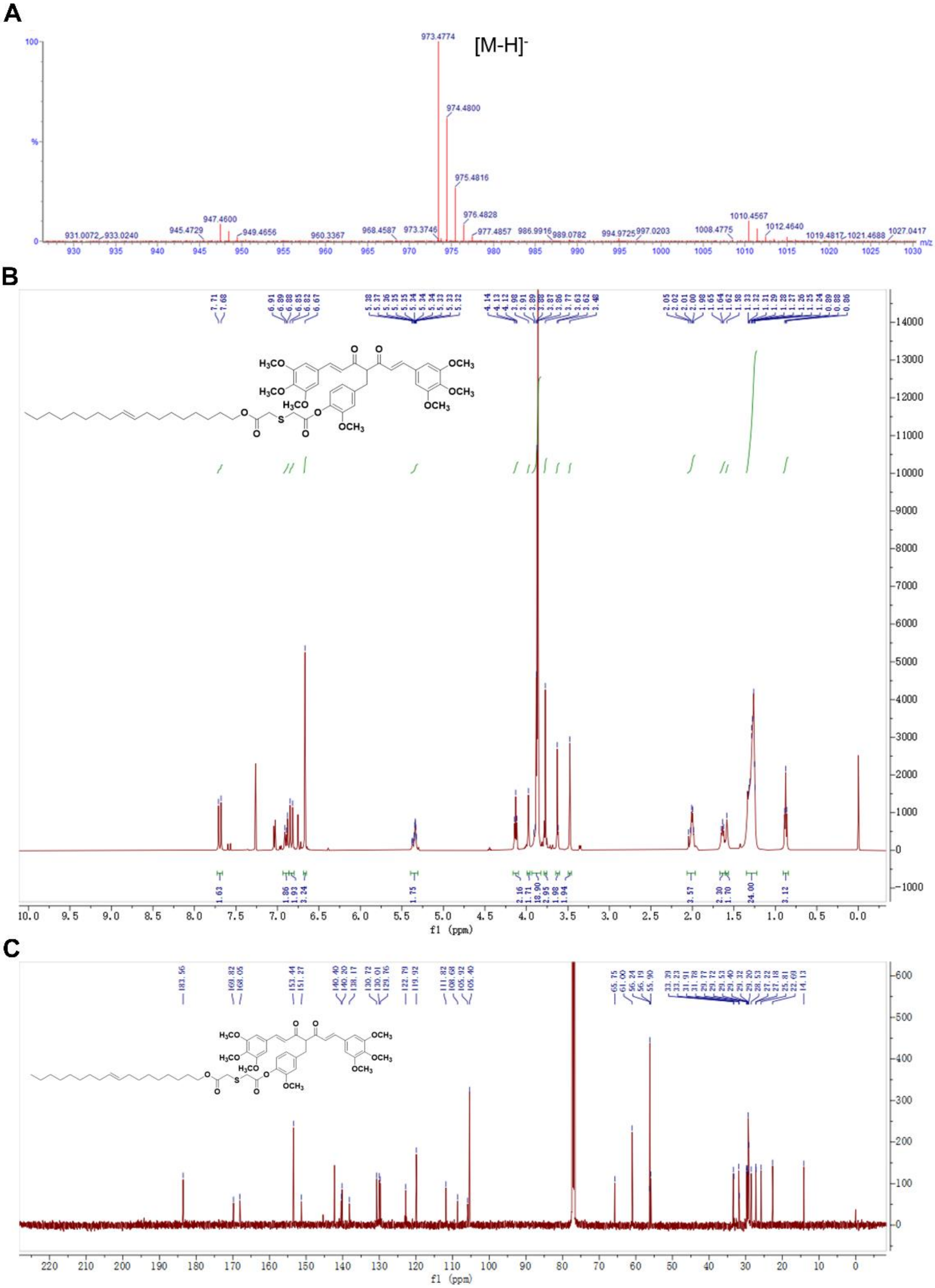

Figure S1. Characterization of C210-S-OA (A) TOF-MS (B) ^{1}H NMR (C) ^{13}C NMR

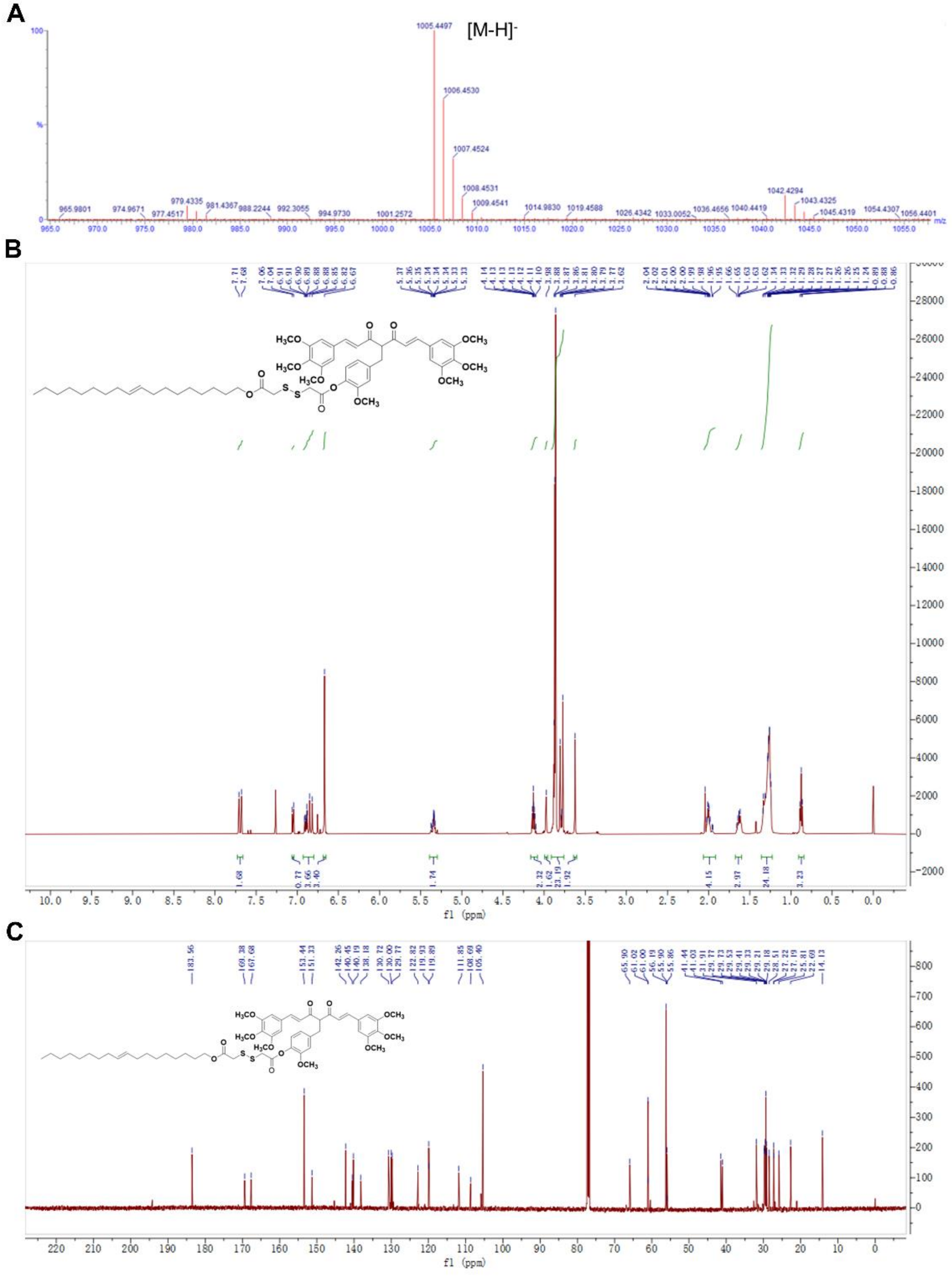

Figure S2. Characterization of C210-SS-OA (A) TOF-MS (B) ^{1}H NMR (C) ^{13}C NMR

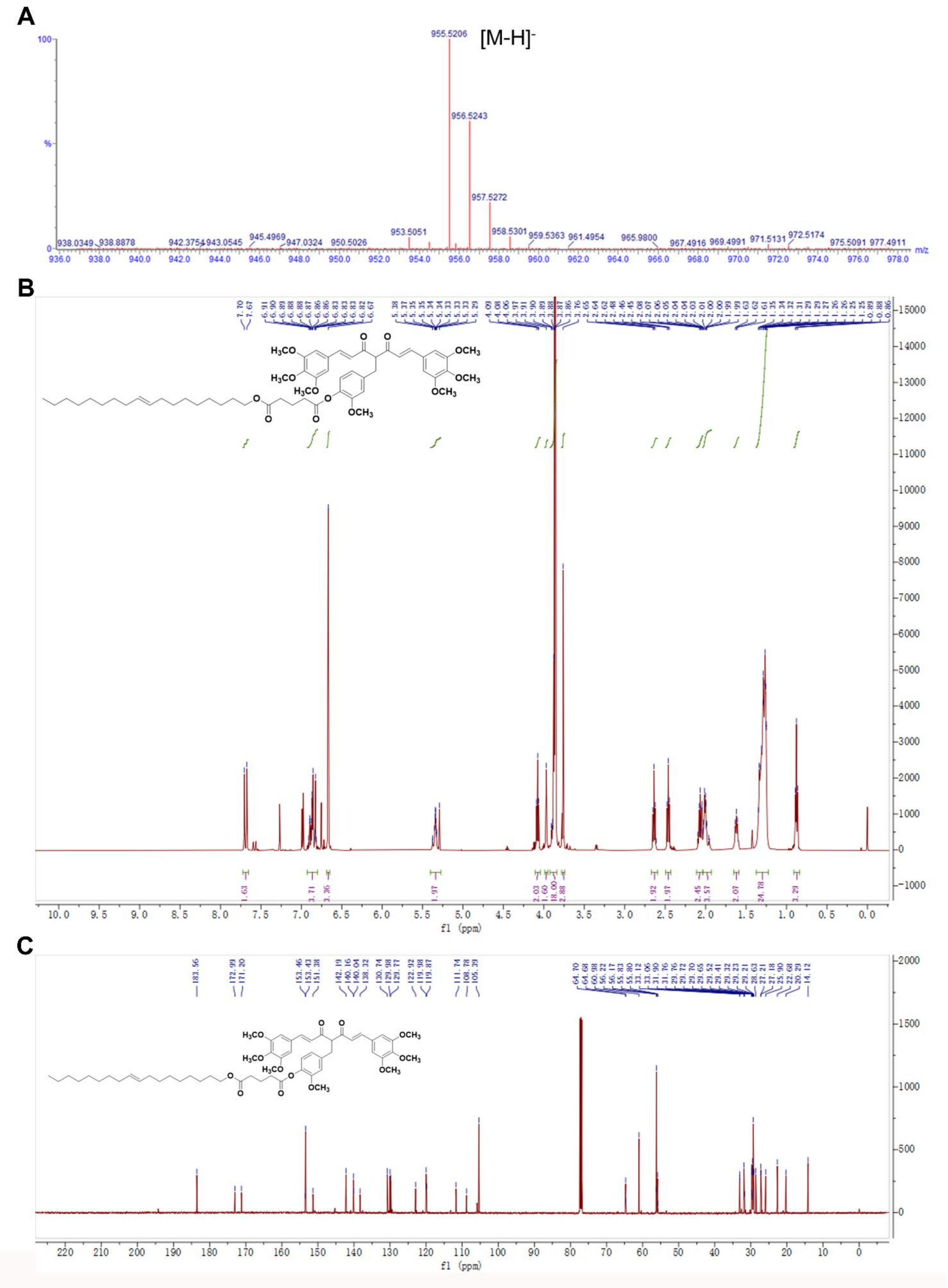

Figure S3. Characterization of C210-C-OA (A) TOF-MS (B) ^{1}H NMR (C) ^{13}C NMR

\

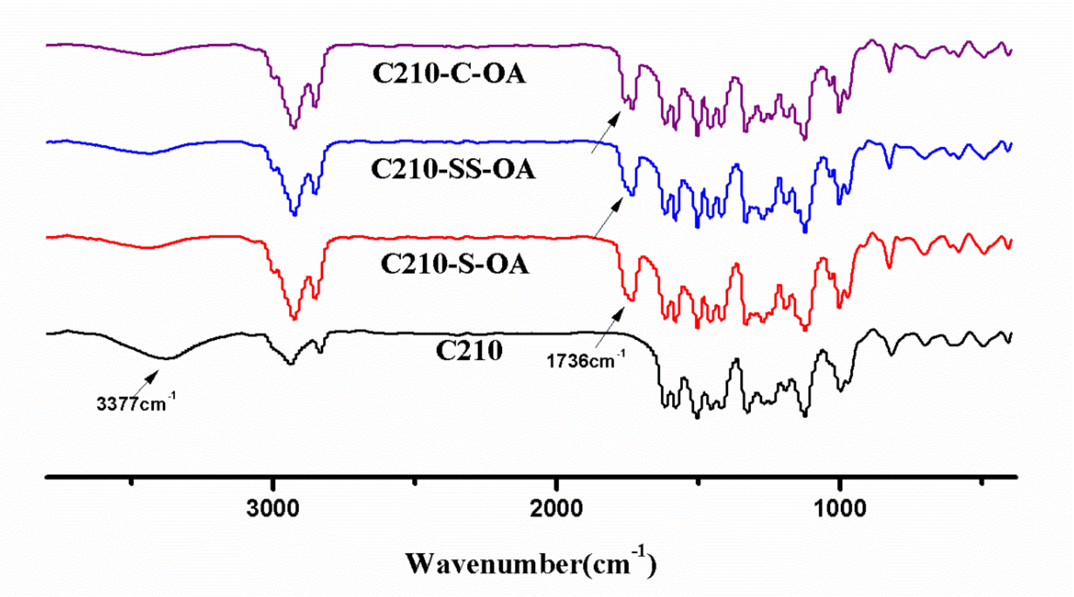

Figure S4. Infrared spectra of C210 and its prodrugs

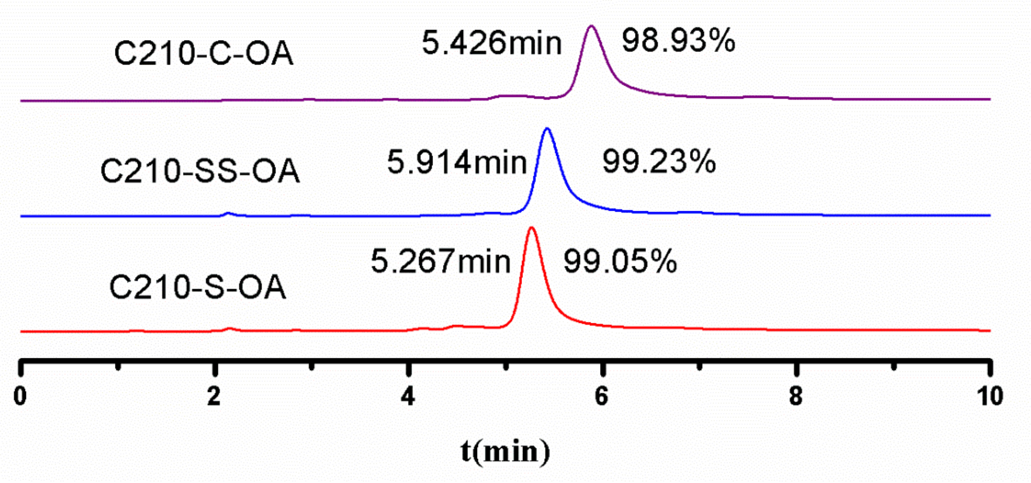

Figure S5. Purity of C210 prodrugs using HPLC

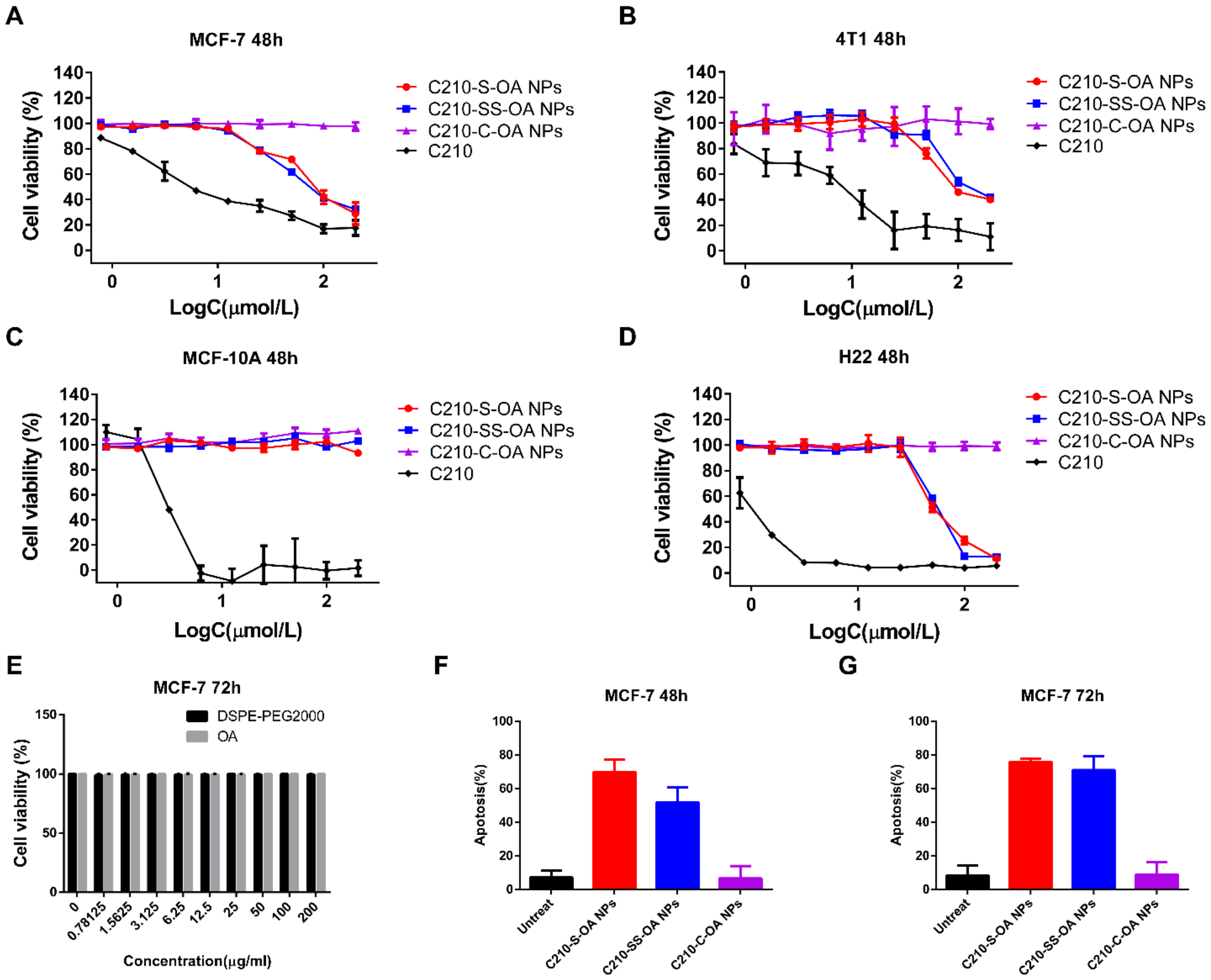

Figure S6. Cell viability and apoptosis induction of C210 prodrugs nanoparticles on tumor cell lines. Cell viability of tumor cells treated with the prodrugs nanoparticles and C210 for 48 h. (A) MCF-7 (B) 4T1 (C) MCF-10A (D) H22 (E) Cytotoxicity assay of DSPE-PEG2k and oleyl alcohol. Apoptosis rate of MCF-7 cells after incubation with different formulations. (F)48 h (G) 72h. Data are presented as mean ± SD (*n*=3).

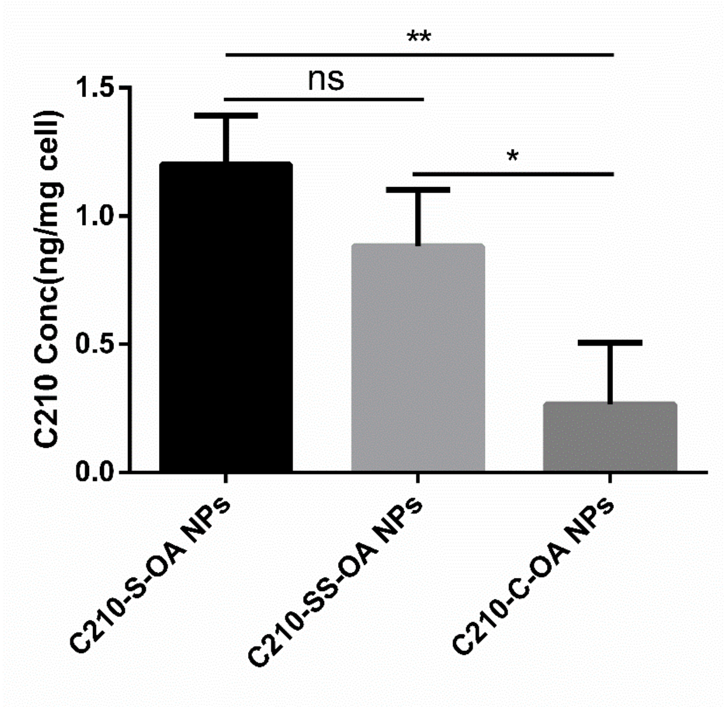

Figure S7. C210 release from prodrugs nanoparticles in MCF-7 cells. Data are presented as mean ± SD (n=3). ***p**＜0.05, **p＜0.01, ns mean not significant.

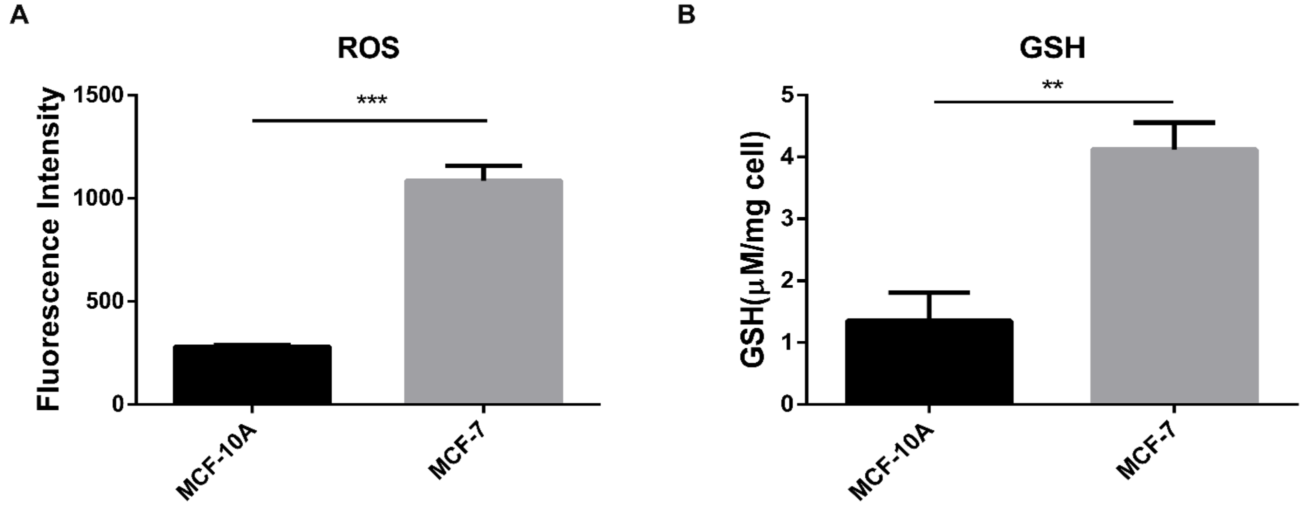

Figure S8. Redox levels between normal cells (MCF-10 A) and tumor cells (MCF-7) (A) ROS (B) GSH. Data are presented as mean ± SD (n=3). **p＜0.01，***p＜0.001.

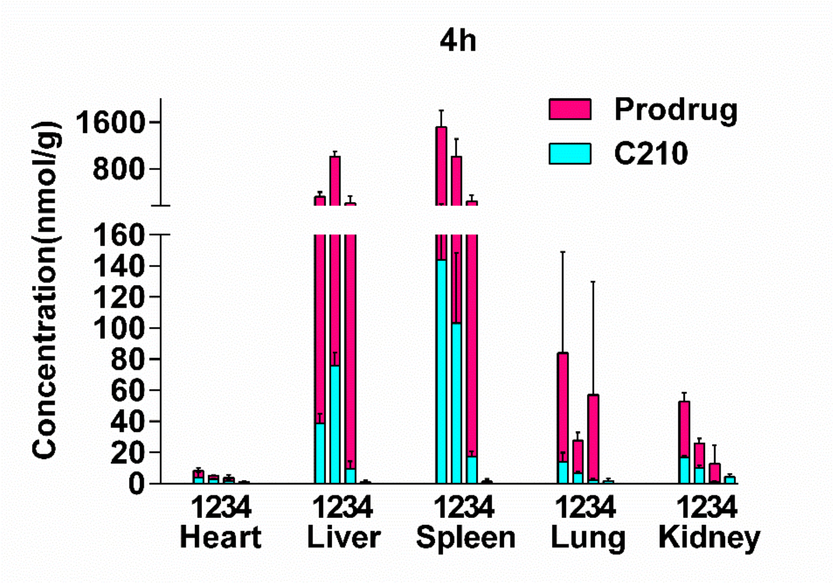

Figure S9. The concentration of each C210 prodrug and its corresponding released C210 in tissues (heart, liver, spleen, lung and kidney) at 4 h after intravenous administration at an equivalent dose of 80 μmol/kg C210 using tumor-bearing BALB/C mice.

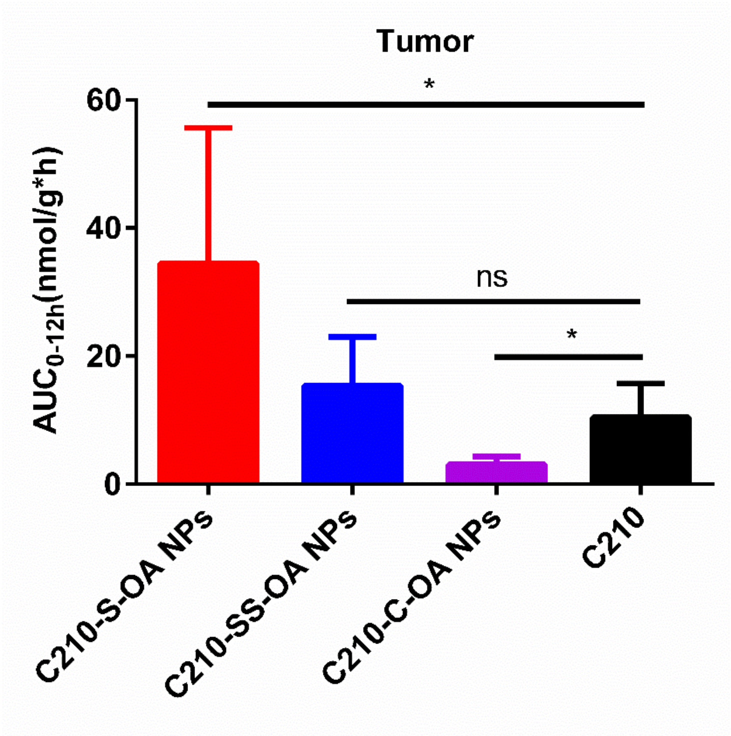

Figure S10. $AUC_{0\text{-}12h}$ of C210 released from C210 prodrugs in tumor tissue after administration. Data are expressed as mean ± SD (*n*=3). *$p < 0.05$, ns mean not significant.

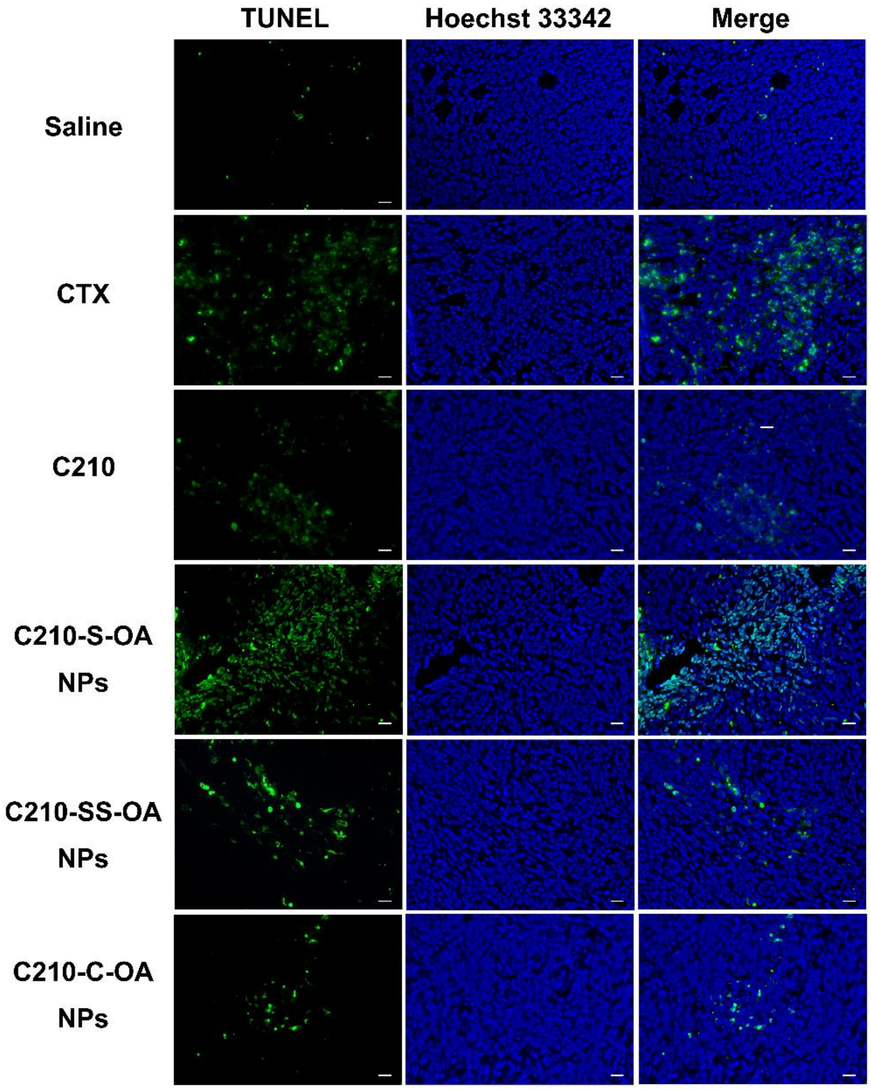

Figure S11. 4T1 tumor section TUNEL assay. Scale bars represent 100 μm.

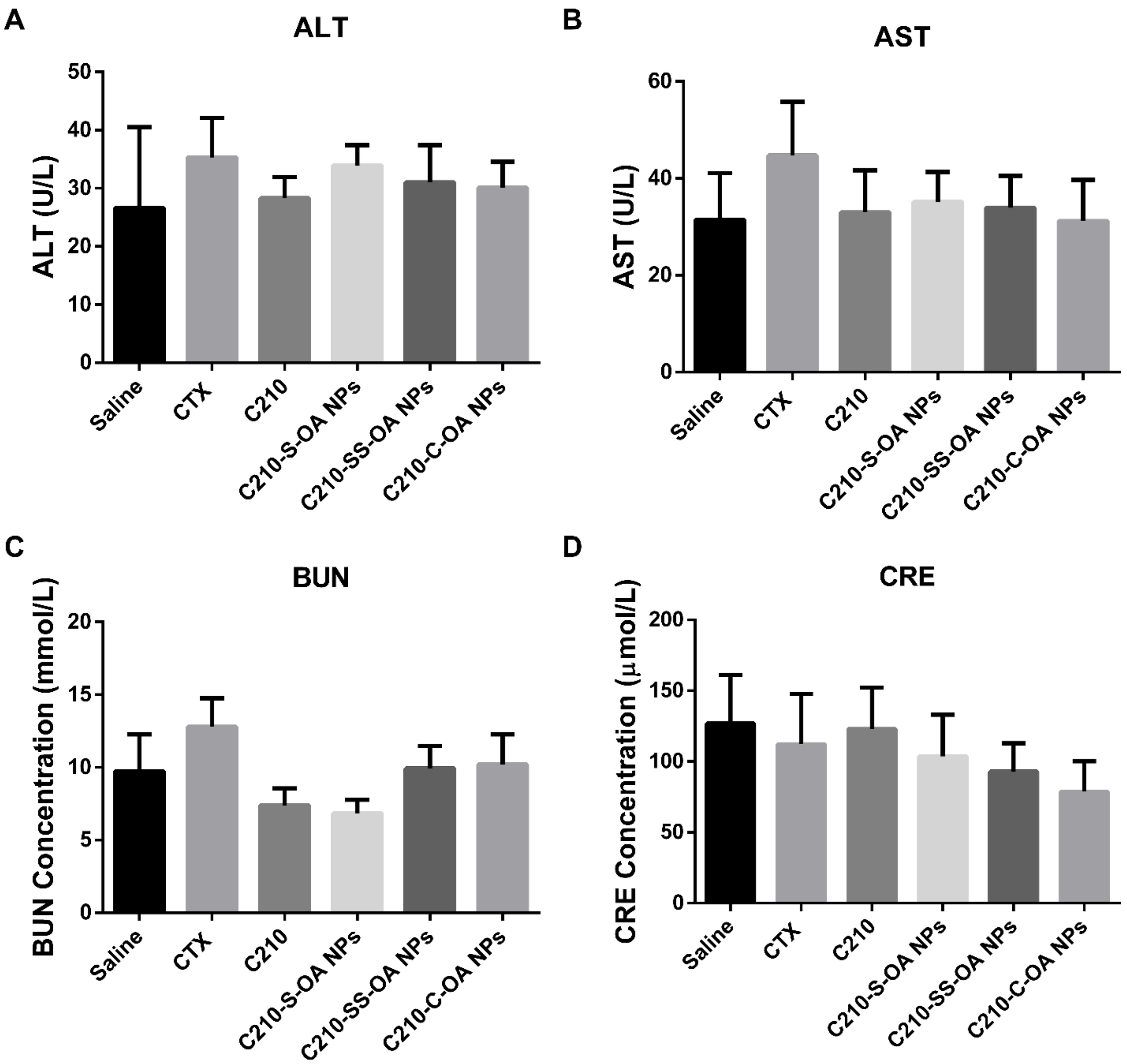

Figure S12. Liver and kidney function parameters of the treated mice (*n*=6). (A) ALT: glutamic aminotransferase (B) AST: glutamic aminotransferase (C) BUN: urea nitrogen (D) CRE: creatinine.

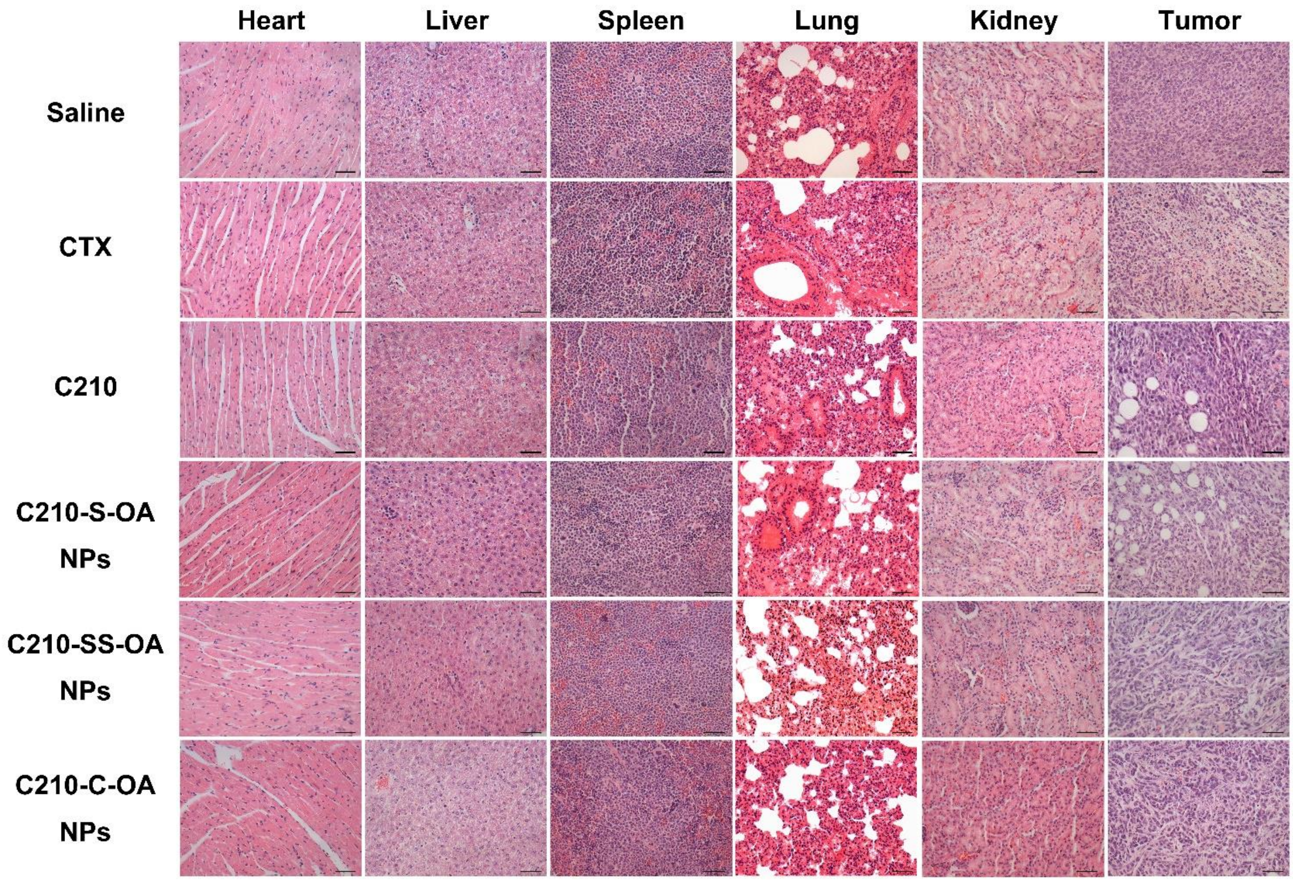

Figure S13. HE-stained images of major organ and tumor tissue sections of treated 4T1 tumor-bearing mice. Scale bars represent 100 μm.

Table S1. Characterization of C210 prodrugs nanoparticles（n=3）

	**Size(nm)**	**PDI**	**Zeta potential(mV)**	**EE(%)**	**DL(%)**
C210-S-OA	128.62±0.84	0.10±0.05	-38.21±1.37	92.39±5.52	51.63±0.48
C210-SS-OA	127.08±0.76	0.10±0.01	-35.29±1.66	98.72±4.68	48.47±0.31
C210-C-OA	122.40±1.63	0.14±0.04	-29.76±2.28	97.93±1.05	49.51±0.31

Table S2. Drug release kinetic models from prodrugs nanoparticles

Condition	Sample	Models	Regression	R^2
H_2O_2	C210-S-OA NPs	Zero-order kinetics	Q=1.10t+38.59	0.44
		First-order kinetics	$Q=82.17(1-e^{-0.35t})$	0.99
		Higuchi	$Q=17.10t^{1/2}+17.86$	0.80
	C210-SS-OA NPs	Zero-order kinetics	Q=2.10t+26.04	0.56
		First-order kinetics	$Q=64.20(1-e^{-0.25t})$	0.98
		Higuchi	$Q=12.96t^{1/2}+11.27$	0.87
	C210-C-OA NPs	Zero-order kinetics	Q=0.41t+2.70	0.65
		First-order kinetics	$Q=11.30(1-e^{-0.14t})$	0.87
		Higuchi	$Q=2.39t^{1/2}+0.20$	0.84
DTT	C210-S-OA NPs	Zero-order kinetics	Q=3.24t+41.75	0.43
		First-order kinetics	$Q=99.55(1-e^{-0.28t})$	0.99
		Higuchi	$Q=21.11t^{1/2}+16.20$	0.79
	C210-SS-OA NPs	Zero-order kinetics	Q=2.22t+65.13	0.09
		First-order kinetics	$Q=97.01(1-e^{-1.94t})$	0.99
		Higuchi	$Q=17.35t^{1/2}+40.57$	0.45
	C210-C-OA NPs	Zero-order kinetics	Q=1.04t+2.32	0.63
		First-order kinetics	$Q=24.48(1-e^{-0.09t})$	0.80
		Higuchi	$Q=5.73t^{1/2}-3.30$	0.72

Table S3. IC_{50} values (μmol/l) of C210 and prodrugs nanoparticles against cancer cells (n=3).

Formulations	4T1		H22	
	**48h**	**72h**	**48h**	**72h**
C210	5.5±1.7	2.8±0.5	1.5±0.2	0.8±0.1
C210-S-OA	92.4±1.2	84.5±9.7	53.5±4.1	37.5±8.4
C210-SS-OA	126.5±21.5	83.8±3.0	69.7±7.3	43.2±3.2
C210-C-OA	＞200	＞200	＞200	＞200

Table S4. IC_{50} values (μmol/l) of C210 and prodrugs nanoparticles between normal cells and cancer cells. Relative therapeutic index =IC_{50} normal /IC_{50} cancer (n=3).

Formulations	MCF-10A		MCF-7		Relative therapeutic index
	**48h**	**72h**	**48h**	**72h**	**72h**
C210	4.7±0.4	1.1±0.2	4.5±0.8	1.4±0.7	0.8
C210-S-OA	＞200	＞200	82.1±1.7	52.5±15.1	＞3.8
C210-SS-OA	＞200	＞200	94.8±3.2	80.5±21.7	＞2.5
C210-C-OA	＞200	＞200	＞200	＞200	-

Table S5. Pharmacokinetic parameters of C210 and C210 released from prodrugs nanoparticles. ***p<0.001 vs. C210 group (n=3).

Formulations	$AUC_{0\text{-}12h}$ (μmol/l*h)	$MRT_{0\text{-}12h}$ (h)	$T_{1/2}$ (h)
C210	74.51±25.50	0.44±0.15	1.17±0.76
C210-S-OA NPs	734.29±106.39***	3.21±0.31***	1.58±0.51
C210-SS-OA NPs	713.72±159.55***	2.97±0.31***	1.79±0.40
C210-C-OA NPs	44.99±18.83	2.50±0.46***	1.32±0.40

Table S6. Pharmacokinetic parameters of C210 prodrugs (*n*=3).

Formulations	$AUC_{0\text{-}12h}$ (μmol/l*h)	$MRT_{0\text{-}12h}$ (h)	$T_{1/2}$ (h)
C210-S-OA NPs	479.95±70.85	2.94±0.42	1.94±0.32
C210-SS-OA NPs	370.02±155.86	3.14±0.32	2.17±1.12
C210-C-OA NPs	508.50±202.57	2.89±0.84	2.37±1.15
